# Copromicroscopic Diagnosis and Prevalence of Parasitic Infections in Animals in Sitio Ibayo, San Mateo, Rizal, Philippines: Establishing a Sentinel Study for Zoonotic Disease Surveillance

**DOI:** 10.7759/cureus.75675

**Published:** 2024-12-13

**Authors:** Ryan V Labana, Rodel Victor D Dimasin, Jacquiline S Tychuaco, Alejandro Jose C Reboa, Armin S Coronado

**Affiliations:** 1 Center for Integrated Community Science Research, Research Institute for Science and Technology, Polytechnic University of the Philippines, Manila, PHL; 2 Department of Biology, College of Science, Polytechnic University of the Philippines, Manila, PHL; 3 Journal Management Section, Research Management Office, Polytechnic University of the Philippines, Manila, PHL; 4 Research Institute for Science and Technology, Polytechnic University of the Philippines, Manila, PHL

**Keywords:** animal health, coproparasite, geohelminthiasis, one health, suburban community

## Abstract

Background: This study investigates the prevalence and intensity of parasitic infections in animal fecal samples collected from Sitio Ibayo, San Mateo, Rizal, Philippines, a suburban community considered a potential sentinel site for zoonotic disease surveillance.

Methods: Using cross-sectional sampling, 132 animal fecal samples were collected in the area exhaustively. Samples were processed through direct smear with saline solution and Lugol’s iodine and flotation technique using mini- and fill-FLOTAC. Microscopy was conducted, and the photomicrographs were analyzed to identify the parasite.

Results: This study revealed an overall prevalence of parasitic infection of 50.67%. The prevalence was 100% in birds, cats, doves, and a rabbit, whereas dogs showed a high prevalence of 68.75%, followed by ducks at 66.67% and humans at 44.44%. Notably, the parasitic infection among ruminants was low, including zero in cows. Farm animals such as pigs and chickens have 53.8% and 42.9% prevalence, respectively. The co-infection was seen as 24 (18.18%) samples had two types of parasites, 12 (9.09%) had three types of parasites, three (2.27%) had four types of parasites, and one (0.76%) had five types of parasites. A total of 17 genera/species of parasites were found, including those belonging to the phylum Nematoda, phylum Platyhelminthes, and phylum Protozoa. Intensity analysis of infections demonstrated high intensity of *Capillaria* spp. infections among 50% of the sampled birds and *Eimeria *spp. among 20% of the sampled geese, with most parasitic infections in the other categories at low intensity.

Conclusion: This study highlights the burden of parasitic infections among various animal groups in Sitio Ibayo. The prevalence and co-infections in companion animals, farm animals, and humans were notable. The high prevalence of parasites in dogs, cats, and birds underscores their potential role in maintaining and dispersing parasitic infections within the ecosystem. The predominance of low-intensity infections suggests that while immediate health impacts may be minimal for some hosts, multi-parasite infections raise concerns for ecosystem health and zoonotic transmission. Targeted interventions using the One Health approach, including improved hygiene practices, deworming programs, and public awareness campaigns, are needed to mitigate the spread of parasitic diseases in this community.

## Introduction

Soil-transmitted helminthiasis (STH) is the most common neglected tropical disease of parasitic origin. It is most prevalent in poverty-stricken areas in developing countries, specifically in tropical and subtropical regions, with approximately 300 million people suffering from heavy STH, resulting in 150,000 deaths annually [1]. In the Philippines, STH is a perennial public health concern due to its high prevalence in urban and rural areas, affecting preschool children (<5 years) and primary school children (5-12 years) [2,3].

In the animal interface, parasitic infection is also a major global concern, affecting livestock, wildlife, and companion animals. Globally, parasitic diseases such as helminthiasis, protozoan infections, and ectoparasite infestations cause substantial economic losses, particularly in agriculture and aquaculture, and pose zoonotic risks [4]. Tropical conditions and diversified ecosystems in the Philippines are conducive to the proliferation of parasites, which are abundant in both domestic and wild animal populations [5]. The common parasitic infections in the country include gastrointestinal nematodes, liver flukes, and blood parasites, affecting livestock production and endangered wildlife species [6-8]. These infections have implications for One Health, considering human, animal, and environmental health are interlinked.

Parasitic infection, either in animals or humans, is closely linked to poverty, inadequate sanitation, and insufficient access to clean water. ​The environment serves as a reservoir of parasites. It also facilitates the incubation and survival of the parasites for extended periods [4,9]. Meanwhile, human activities such as land-use changes and improper waste disposal increase the exposure of both animals and humans to parasitic infections. This environmental interplay underlines the interconnectedness of ecosystems and health, thus demanding sustainable practices and integrated interventions to reduce the burden of parasitism and its associated risks [10].​

A sentinel site for zoonotic disease surveillance: Sitio Ibayo, San Mateo, Rizal Philippines

Sitio Ibayo, Brgy. Dulong Bayan, San Mateo, is a low-lying, first-class municipality in Rizal, Philippines. It is bordered by Quezon City to the west, the cities of Marikina and Antipolo to the south, and the municipality of Rodriguez to the north. It is located between Brgy. Dulong Bayan I and Brgy. Dulong Bayan II. The whole Dulong Bayan has an approximate land area of 510.085 sqm with a total population of 15,721. Rivers and creeks predominantly move from east to west, following the contour of the area that slopes westward to the Marikina Valley [11]. It is characterized by limited water and sanitation services. Deep wells and other groundwater resources are the main source of the municipality's water supply. Access to Sitio Ibayo is complicated by the Marikina River, which traverses the main entryway, making it difficult for residents and San Mateo, Rizal's local government, to reach the community [12]. These geographical and logistical challenges have hindered overall development in the area, amplifying the community's vulnerabilities to various public health problems, including parasitic infections.

A high interaction between humans and animals is evident in the area, inhabited by various free-ranging animals, including ruminants, companion animals, and birds. In 2020, an unpublished undergraduate thesis from the Polytechnic University of the Philippines reported a high prevalence rate (~70%) of STH among children. This data and other factors, including the presence of free-ranging animals, the hygiene and sanitation condition of the area, and population characteristics, make Sitio Ibayo a potential sentinel site for understanding the transmission dynamics of zoonotic disease in a suburban setting. This paper analyzes the prevalence and intensity of parasitic infections among the animal groups in the area. Understanding these factors is crucial for assessing veterinary health and the potential for zoonotic transmission of parasitic diseases.

## Materials and methods

Research design

This study used a cross-sectional design. Data analysis followed a mix of descriptive, diagnostic, and correlational techniques. The prevalence rate of parasitic infection was associated with various factors, including polyparasitism, parasite diversity, intensity of infections, and hosts.

Sampling sites and techniques

This study was conducted during the dry season from January to May 2024 in an underserved suburban community in Sitio Ibayo, Dulong Bayan I, San Mateo, Rizal, Philippines (14°42'05.8 "N 121°07'23.2" E). The place serves as a transitional zone between urban and rural areas. It is characterized by residential areas, small-scale farms, and pockets of undeveloped land, providing a diverse habitat for domestic and peridomestic animals.

**Figure 1:**
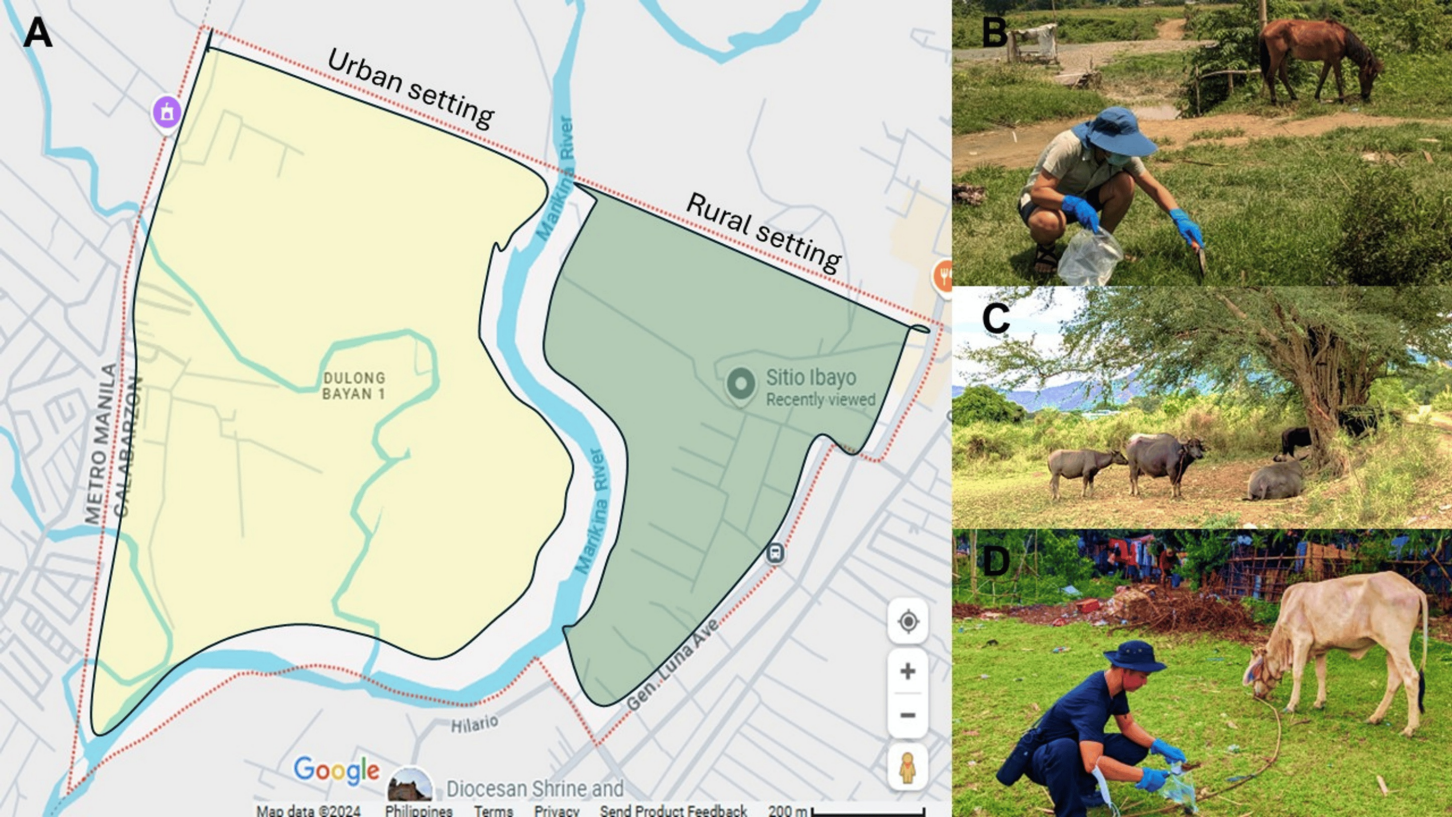
The research site (A) Map showing the urban and rural settings in Sitio Ibayo, with annotations added by the author to highlight the key areas; (B) fecal sample collection in the farm area; (C) the area where ruminants are taking a rest; (D) fecal sample collection in the residence area Screenshot and annotations by Ryan V. Labana Map data ©2024 Google [12]

The fecal samples were collected from the environment in Sitio Ibayo. The map is highlighted green in Figure 1. The animal source of the feces was determined by the distinct appearance of each type. For example, goat feces are characterized by small, round, pellet-like droppings that are typically dark brown to black. On the other hand, carabao feces appear as large, flattened piles that are dark green to brown, while bird feces have a soft and moist consistency due to the combination of feces and urates. In most cases, the animal source of the fecal samples was confirmed by proximity to the feces. For instance, Figures 1B, 1D show the presence of horses and cows at the collection sites, respectively. Additionally, the freshness of the feces, characterized by a moist and shiny surface, further confirms that it came from an animal source nearby.

The collection of the fecal samples was conducted exhaustively. The area was divided into four sites to ensure a systematic and organized sample collection. Site 1 comprises open fields primarily used as grazing areas for ruminants. Sites 2 and 3 represent the residential zones within the barangay, where high human activities were observed. Site 4, like Site 1 in an open area, is notable for its high population density of various animals. Using a spatula, all feces observed within the vicinity of the barangay were obtained and placed in clean, labeled, resealable plastic bags. All samples were collected from surfaces not in contact with the substratum to avoid contamination [7]. The fecal samples were stored in a container during transport to the laboratory and processed within 48 hours of collection.

Detection of parasites in the animal fecal samples

Animal fecal samples were subjected to microscopic examinations to detect parasitic ova, cysts, larvae, and adult forms. The direct smear and flotation techniques using mini- and fill-FLOTAC were employed. In the direct smear method, a thin smear of fecal material mixed with either saline or Lugol's iodine was prepared on a glass slide and examined under a light microscope, enabling preliminary analysis of the fecal samples [3]. The findings of this method were used to complement the results of the mini- and fill-FLOTAC technique, which concentrated parasitic elements by suspending the fecal material in a high-density (1.3 specific gravity) sucrose solution, allowing lighter parasitic stages to float to the surface for detailed microscopic examination [13].

Microscopic identification of parasites

Morphometric and qualitative analyses were performed on photographs of the observed parasites under the microscope. Parasites were identified based on their morphological characteristics using standard keys and descriptions. Resources from the Centers for Disease Control and Prevention (CDC) [14] and other available literature on the morphology of intestinal parasites in humans and animals, including the fourth edition of Veterinary Parasitology by Taylor, Coop, and Wall [15], were used as guidelines for parasitological identification in the study. Photomicrographs of the identified parasites were taken for record-keeping and further analysis.

Three researchers thoroughly identified the parasites. They compared the morphology of the parasite eggs with published literature [14,15], focusing on key morphometric data. Table 1 presents the different parasites detected in the animal fecal samples collected from Sitio Ibayo, along with the morphological characteristics of the parasites examined through photomicrographs and direct observations under the microscope, as well as the references for the sources.

**Table 1:** Morphological characteristics of the eggs, ova, or cysts used in this study in the identification of parasites

Parasites	Morphological Features	Reference
*Ascaridia *spp.	The eggs are elliptical, thick-walled, and colorless, with a granular brownish content, about 80×50 µm in size.	[14,15]
*Ascaris* spp.	The fertile eggs range from 45 to 75 µm in length. Unfertilized eggs are elongated and more prominent than fertile eggs (up to 90 µm in length). Their shell is thinner, and their mamillated layer is more variable.	[14,15]
*Capillaria* spp.	The eggs are 50–70 µm long x 30–35 µm wide. They have a striated shell and shallow polar prominences. They may resemble *Trichuris* spp. eggs but are more ellipsoid than barrel-shaped, with smooth instead of protruding poles.	[14,15]
*Clonorchis* spp.	The eggs are small, ranging from 27 to 35 µm x 11 to 20 µm. They are oval-shaped with a convex operculum that rests on visible "shoulders" at the smaller end. At the opposite (abopercular) end, a small knob or hook-like protrusion is often visible.	[14,15]
*Eimeria* spp.	The oocysts are oval- to egg-shaped without micropyle and micropylar caps and with double-layered oocyst walls.	[15]
*Entamoeba* spp.	The cysts have 4 nuclei with centrally located karyosomes and fine, uniformly distributed peripheral chromatin. Cysts usually measure 12–15 µm.	[14,15]
Giardia lamblia	The cysts are oval to ellipsoid, measuring 8–19 µm, with an average size of 10–14 µm. Mature cysts contain four nuclei, whereas immature cysts contain two. Both iodine-stained wet mounts and trichrome-stained smears reveal visible nuclei and fibrils.	[14,15]
Hookworm	The eggs are thin-shelled and colorless, measuring 60–75 µm x 35–40 µm.	[14,15]
*Isospora* spp.	The oocysts display a distinctive 1:2:4 configuration, with each oocyst containing 2 sporocysts, each of which houses 4 sporozoites. These oocysts are typically ovoid to ellipsoid, measuring 10–40 µm in length and 10–30 µm in width. They may also contain specialized structures, including polar caps, micropyles, residual bodies, and crystalline bodies.	[14,15]
*Parascaris* spp.	Eggs are brownish, subspherical, and have a thick, finely punctated shell that measures 90–100 µm in diameter. When expelled in feces, each egg contains a single cell.	[15]
*Passalurus* spp	The eggs typically have a flat side and measure about 100x43 mm.	[15]
*Spirometra* spp.	The eggs of *Spirometra* spp. are operculate, ovoid, and measure 55–76 x 30–43 µm.	[15]
*Strongyloides* spp. (egg)	The eggs are slightly ovoid, measuring 50–60 x 30–40 µm, with a thin, colorless shell, and are excreted partially embryonated.	[14,15]
*Strongyloides* spp. (larvae)	The first-stage rhabditiform larvae (L1) measure 180–380 µm in length. They feature a short buccal canal and a rhabditoid esophagus, divided into three sections extending to one-third of the body length. Additionally, they possess a prominent genital primordium. In contrast, the second-stage rhabditiform larvae (L2) are longer and exhibit a smaller esophagus-to-intestine ratio.	[14,15]
*Taenia* spp.	The eggs measure 30–35 micrometers in diameter and are radially striated. The internal oncosphere contains six refractile hooks.	[14,15]
*Toxocara* spp.	The eggs are golden in color, spherical to slightly pear-shaped, thick-shelled, and have a pitted surface. The size range for different species varies slightly; *T. canis* is larger (80–85 µm) than *T. cati* (65–75 µm).	[14,15]
Trichuris trichiura	The eggs measure 50–55 µm x 20–25 µm. They are barrel-shaped, thick-shelled, and have a pair of polar "plugs" at each end.	[14,15]

Positive specimens were not submitted to the third-party assessor for validation. Instead, a systematic validation among three parasitology researchers was conducted, wherein specimens were individually identified by two researchers based on the established guides from the literature [14,15]. Any disagreement on the identification of the parasite was mitigated by another parasitology researcher.

## Results

Animal groups and their parasitic infections

A total of 132 fecal samples were collected from the environment of Sitio Ibayo. Of these samples, one (0.76%) was from a rabbit, two (1.52%) from birds and doves, three (2.27%) from ducks and cats, five (3.79%) from geese, seven (5.30%) from chickens and goats, nine (6.82%) from humans and carabaos, 11 (8.33%) from horses, 12 (9.09%) from cows, 13 (9.85%) from pigs, and 48 (36.36%) from dogs. Birds and doves are presented separately because doves were explicitly identified by their owners, while the two fecal samples from unidentified birds were collected from the bird droppings trap in the cornfield. This presentation also includes human samples collected from the environment rather than households. The human fecal samples were specifically identified by the inhabitants of Sitio Ibayo.

**Figure 2:**
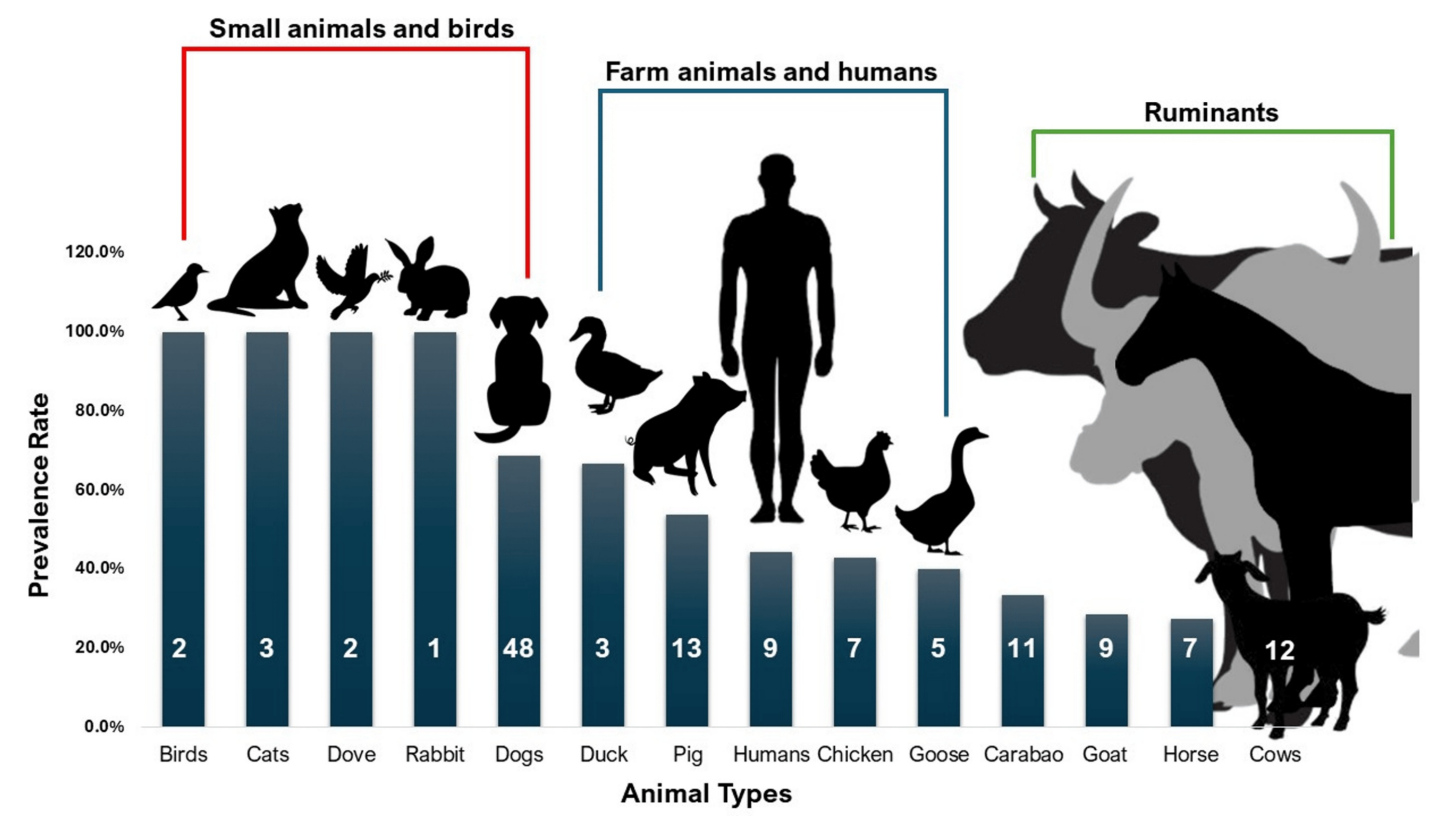
Prevalence rates of parasitic infection with at least one type of parasite among various animal groups. The number inside each bar is the number of samples collected from each animal group Image Credits: Ryan V. Labana

The overall prevalence of parasitic infection across different types of animals is 50.67%. Figure 2 presents the prevalence rates of parasitic infection by animals. High prevalence rates were observed among birds, cats, doves, and rabbits. All samples collected from these animals tested positive for at least one type of parasite. Of the 48 fecal samples from dogs, 33 (68.75%) were positive for at least one type of parasite. The prevalence rates of parasitic infection among other animal groups are as follows: n=1/3; 66.67% in ducks, n=4/9; 44.44% in humans, n=3/7; 42.86% in chickens, n=2/5; 40.00% in geese, n=3/11; 27.27% in horses, n=3/9; 33.33% in carabaos, n=2/7; 28.57% in goats, and nil in cows. A pattern is observable among animal groups based on the prevalence rates of parasitic infections. The highest prevalence rates were observed in small animals and birds, while the lowest, including 0%, were observed among ruminants. Farm animals and humans had prevalence rates ranging from 40% to 66.67%.

**Figure 3:**
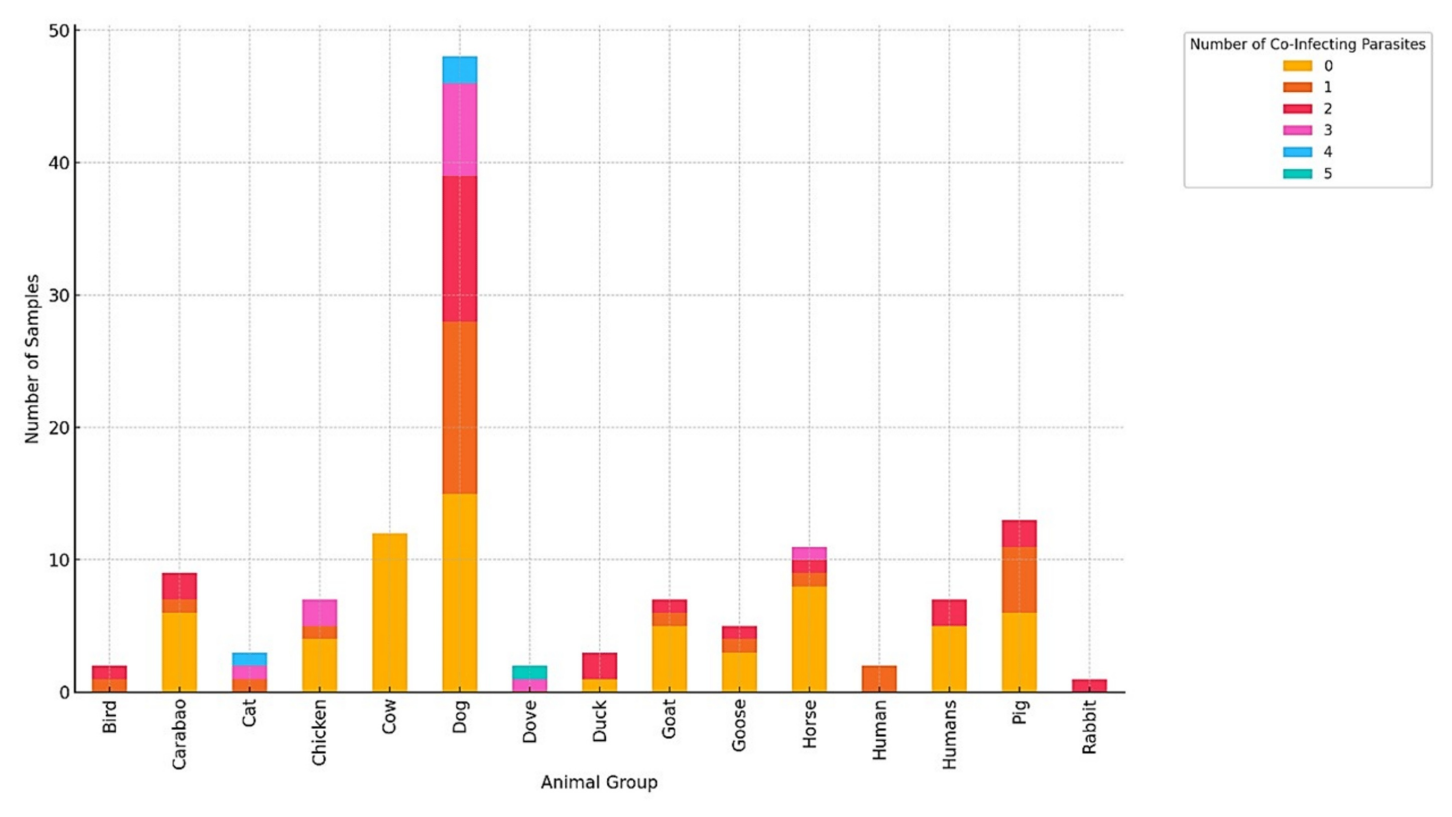
Co-infection patterns per animal group

Further analysis shows that parasitism in many animals is caused by multiple parasites. Of the 132 fecal samples, 65 (49.2%) had no parasites, 27 (20.45%) had one type of parasite detected in each specimen, 24 (18.18%) had two types of parasites, 12 (9.09%) had three types of parasites, three (2.27%) had four types of parasites, and one (0.76%) had five types of parasites. Figure 3 presents the co-infection patterns per animal group. Dogs had the highest co-infections, with multiple samples infected by three or more parasites. In contrast, birds and humans have minimal co-infections. In most animal groups, samples infected by no parasite or only one parasite are common. However, dogs and pigs have a more extended distribution, with many multi-parasite infections. Dogs and cats are the major carriers of many parasites, suggesting their possible role in maintaining and dispersing co-infection within ecosystems.

Parasites detected from various animal fecal samples

**Figure 4:**
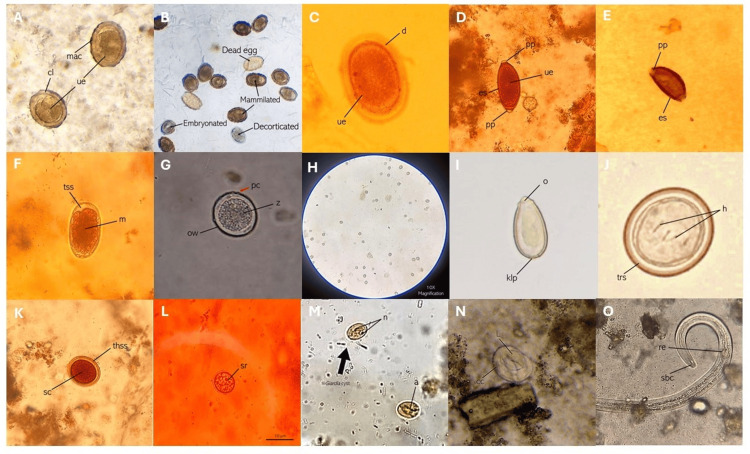
Photomicrographs of the parasites detected from the various animal groups in Sitio Ibayo, San Mateo, Rizal (A) *Ascaris lumbricoides* eggs; (B) different stages of *A. lumbricoides* eggs; (C) Ascarid egg; (D) *Trichuris trichiura* egg; (E) *Capillaria* spp. egg; (F) hookworm egg; (G) *Eimeria* spp. egg; (H) *Eimeria* spp. eggs at 10× magnification; (I) *Clonorchis* spp. eggs; (J) *Taenia* spp. egg; (K) *Toxocara* spp. egg; (L) *Entameoba* spp. egg; (M) *Giardia* spp. eggs; (N) *Strongyloides* spp. egg; (O) *Strongyloides* spp. larvae a: axonemes; cc: chitinous cortex; cl: chitinous layer; h: hook; klp: knob-like projection; l: larvae; n: nucleus; mac: mammilated albuminous covering; m: morula; o: operculum; ow: oocyst wall; pc: polar cap; pp: polar plugs; re: rhabditoid esophagus; sc: single cell; sbc: short buccal canal; sr: sporocyst residuum; thss: thick smooth shell; trs: thick radiate shell; tss: thin smooth shell; ue: undeveloped embryo; z: zygote Image Credits: Ryan V. Labana

A total of 17 parasite species and one unidentified coccidia were detected in the animal fecal samples collected from Sitio Ibayo, San Mateo, Rizal. The photomicrographs of some species are presented in Figure 4. In the group of phylum Nematoda (roundworms), the following parasites were detected: *Ascaridia* sp., *Ascaris lumbricoides*, *Ascaris* spp. detected from pigs, *Toxocara* spp., *Passalurus* spp., *Parascaris* spp., *Trichuris trichiura*, *Capillaria* spp., *Strongyloides* spp., and hookworm. In the group of flatworms under the phylum Platyhelminthes, *Clonorchis spp., Spirometra* spp., and *Taenia* spp. were detected. Other parasites include *Giardia *spp., a flagellated protozoan; *Entamoeba* spp. under the phylum Amoebozoa; and the coccidian protozoans, *Eimeria* spp. and *Isospora* spp.

Prevalence and intensity of parasitic infections

**Figure 5:**
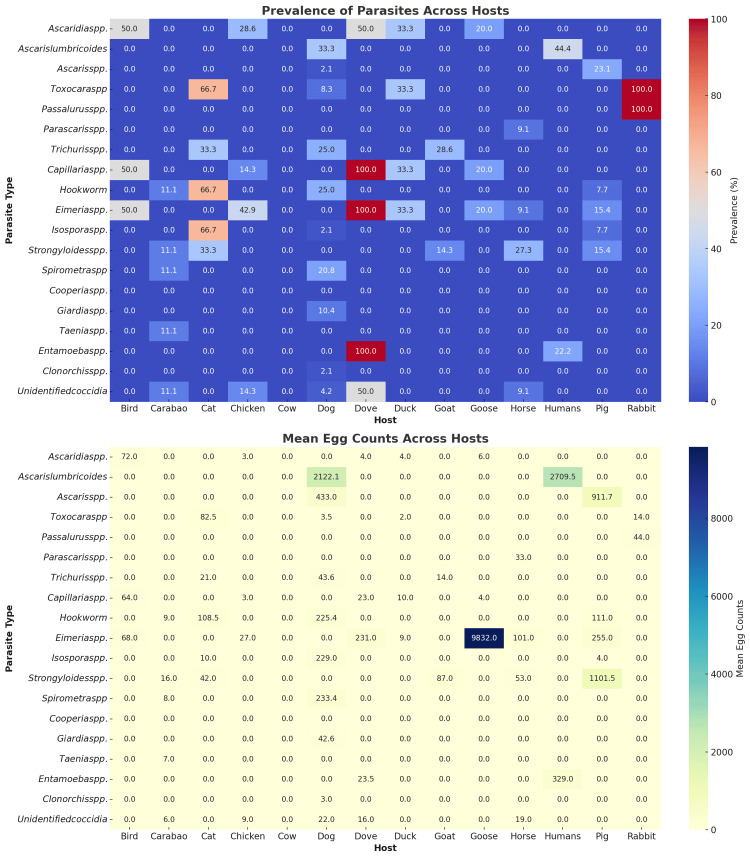
Prevalence of parasitic infections and the mean egg counts across hosts and parasites

The comprehensive overview of the prevalence and egg count of parasitic infections per animal host is presented in Figure 5. Interestingly, three parasites are present in all sampled doves (n=2), implying three parasitic infections with 100% prevalence rates. These include infections with *Capillaria* spp., *Eimeria* spp., and *Entamoeba *spp. However, the intensity of these infections differs, with *Eimeria* spp. having the highest egg count of 231 eggs per gram (EPG). *Capillaria* spp. and *Entamoeba* spp. both have ~23 EPG. One fecal sample from a rabbit was analyzed, and it was positive for both *Toxocara* spp. and *Passalurus* spp.

Other key findings include the high prevalence rates for different parasites in dogs and cats. Dogs are infected with several parasites, including *Ascaris lumbricoides*,* *at a 33.3% prevalence rate. Other parasites detected from dogs include *Trichuris* spp. (25%), hookworm (25%), and *Spirometra* spp. (20.8%). Cats also have parasitism, which encompasses several parasites, including *Toxocara* spp. (66.67%), hookworm (66.67%), *Isospora* spp. (66.67%), *Trichuris trichiura* (33.33%), and *Strongyloides* spp. (33.33%).

Like chickens and pigs, livestock share the same type of parasite, with a high prevalence of *Eimeria* spp. at 42.9% in chickens and 15.4% in pigs. Meanwhile, *Ascaridia* spp. was shared by birds (50%), doves (50%), ducks (33.3%), chickens (28.6%), and geese (20%).

The intensity of infection was analyzed by the animal group because the thresholds for low, medium, and high infection intensity, based on EPG values, vary among species. The World Health Organization has established guidelines for analyzing infection intensity for *Ascaris* spp., *Trichuris* spp., and hookworms. For *Ascaris* spp., low intensity is defined as 1-4,999 EPG, medium intensity as 5,000-49,999 EPG, and high intensity as ≥50,000 EPG. For hookworm infections, the thresholds are 1-1,999 EPG for low intensity, 2,000-3,999 EPG for medium intensity, and ≥4,000 EPG for high intensity. For *Trichuris* spp., low intensity is classified as 1-999 EPG, medium as 1,000-9,999 EPG, and high intensity as ≥10,000 EPG. These thresholds are typically used to diagnose human parasitic infections caused by soil-transmitted helminths and are not necessarily the thresholds for the same infection with the animals [16].

While most parasites lack standardized thresholds for classifying infection intensity, some studies have reported specific values. These thresholds can be influenced by the host type, detection method, and geographic region of parasitism. In this study, we adhered to the thresholds established in the published literature. *Capillaria* spp.'s thresholds are 1-10 EPG for low intensity, 11-49 EPG for medium, and ≥50 EPG for high intensity [17]. *Eimeria* spp. has thresholds of <1,000 EPG for low intensity, 1,000-5,000 EPG for medium, and >5,000 EPG for high intensity [18]. *Isospora* spp.'s thresholds are <500 EPG for low intensity, 501-999 EPG for medium, and >1,000 EPG for high intensity [19]. *Parascaris* spp. has a threshold of <200 EPG for low intensity, 200-999 EPG for medium, and >1,000 EPG for high intensity [20]. *Clonorchis* spp. has thresholds of <1,000 EPG for low intensity, 1,000-9,999 EPG for medium, and >10,000 EPG for high intensity [21].

No published reports on the threshold were found for other parasites, including *Ascaridia* spp., *Toxocara* spp., *Taenia* spp., *Strongyloides* spp., *Spirometra* spp., *Passalurus* spp., *Entamoeba* spp., and *Giardia* spp. Without established thresholds for classifying parasite intensity levels, a presence-based classification approach was adopted. For parasites without defined criteria for "low" or "high intensity," any detected non-zero parasite counts were defaulted to "low intensity." This method assumes that the mere presence of the parasite indicates a minimal level of infection, justifying the classification as low intensity. By adopting this approach, we ensure that all recorded data are included while acknowledging the limitations caused by the lack of standard intensity thresholds. This framework facilitates consistent data interpretation and emphasizes the need for further research to establish precise thresholds for these parasites [22].

**Figure 6:**
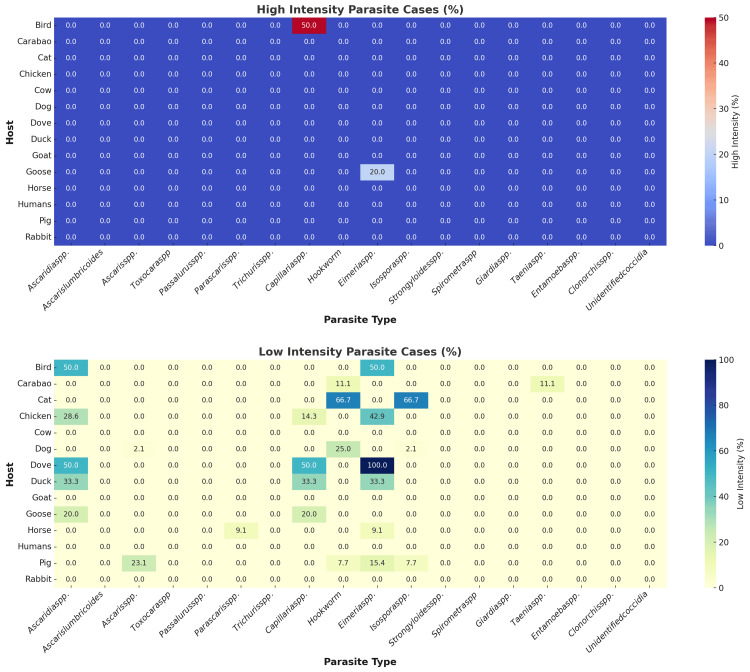
Intensity of parasitic infections by host and parasite type

Figure 6 presents the intensity of parasitic infections by host and parasite type. It is observable that 50% of sampled birds have high-intensity *Capillaria* infections, while 20% of the sampled geese have high-intensity *Eimeria* infections. The majority of parasitic infections have low intensity.

## Discussion

Implications on veterinary health and animal management

The parasitic infections varied among the diverse animal species within Sitio Ibayo, San Mateo, Rizal, Philippines, a suburban community. Prevalence rates were high among birds, cats, and dogs, although at different percentages. It is shown that 100% of the birds and cats are infected with at least one parasite. The results can be attributed to the frequent contact with contaminated environments such as soil and water sources and the lack of deworming and veterinary care among these animals. The dogs were the most sampled group (36.36% of the total samples), showing a prevalence rate of 68.75%, underlining their important role in sustaining populations of parasites.

Farm animals, a very integral part of the livelihoods in Sitio Ibayo, had parasitism at an intermediate prevalence rate. For instance, geese and chickens had prevalence rates of 40.00% and 42.86%, respectively. The susceptibility of farm animals to parasitism is caused by several factors, including manure accumulation and environmental exposure to open areas for grazing and drinking in potentially parasite-contaminated areas [23]. Similarly, pigs showed a prevalence rate of 66.67%, indicating susceptibility to multiple parasites in shared environments with other animals and humans. In contrast, higher-level ruminants like cows and horses showed a significantly lower prevalence rate, with the rates being 0% and 27.27%, respectively. This pattern may show their grazing behavior in less contaminated areas or be due to greater resilience to parasitic infection because of host-specific immunity [24]. The possible routine deworming with ruminants before the fecal sampling may have also caused this trend.

The findings from the study have strong implications regarding veterinary health and animal husbandry, particularly concerning small animals, farm animals, and birds. High prevalence in small animals such as dogs, cats, and poultry requires frequent deworming programs and a strict standard of hygiene in preventing and controlling infections. Some of these parasites, for instance, in pigs and chickens, share other parasites such as *Eimeria* spp. and *Ascaridia* spp., which require targeted management strategies that include rotational grazing and proper sanitation to minimize environmental contamination. The high prevalence of multi-parasite infections among dogs and pigs highlights the need for integrated parasite control programs aimed at animal groups. Such programs should also consider the threat of co-infections, which may worsen animal health problems and even further decrease productivity in farm animals and undermine the health of companion animals.

Possible transmission routes and sharing of parasites among animals

Direct contact, contaminated environments, and shared resources such as water or grazing areas are likely conduits by which parasite sharing occurs in animal groups [7,24,25]. Dogs and cats, serving as key reservoirs of many species of parasites, may play a central role in maintaining and distributing infection within ecosystems. For example, intense infections of *A. lumbricoides* and *Toxocara* spp. in dogs and cats indicate potential contamination of environmental resources through fecal inputs. Shared parasites like *Ascaridia* spp. in birds, ducks, and geese indicate the need for a common route of transmission, which is often a shared nesting or feeding ground. Some ways to reduce interspecies transmission are isolating infected animals, ensuring proper waste management systems, and using species-specific feeding and housing facilities.

Additionally, *A. lumbricoides* was detected in 33.33% of the dog fecal samples. It is an intestinal nematode species generally implicated with human infections, which was also detected in humans in this study with a prevalence of 44.44%. This shows that *A. lumbricoides* in dogs can reflect cross-species transmission, or environmental contamination could be shared. In general, *A. lumbricoides* is more of a human parasite. Its presence in dogs raises the possibility that it acts as a paratenic host or contributes to contamination within an environment with viable eggs. This finding emphasizes the close interaction between humans and dogs in Sitio Ibayo and highlights the necessity for further research to determine whether dogs can be true hosts for this parasite or only carry eggs from the environment. It also implies that humans and animals in Sitio Ibayo are creating a cycle of infection due to their exposure to contaminated soil, water, or surfaces, perpetuating the prevalence of the parasite.

Zoonotic transmission and the One Health approach

Most of the parasitic infections identified in this study are caused by zoonotic parasites, including *A. lumbricoides,* *Giardia* spp., and *Entamoeba* spp., pointing toward public health hazards. Additionally, *Giardia* spp., mainly acquired through infected water sources, indicates that sanitation is an issue in this locality. The detection of *Clonorchis* spp., a trematode associated with fishborne zoonotic diseases, raises concerns about the safety of local water bodies as its life cycle may be promoted by fecal contamination by humans and companion animals such as dogs and cats [26]. Environmental contamination with fecal matter from multiple hosts is a significant driver of parasite persistence and transmission.

In Sitio Ibayo, a targeted One Health approach can address the high prevalence of parasitic infections such as *A. lumbricoides* by integrating human, animal, and environmental health strategies [27]. First, continuous assessment and monitoring should be conducted using parasitological surveys and molecular techniques to identify parasites' prevalence, intensity, and transmission dynamics in humans and animals. This would clarify the role of dogs in the lifecycle of *A. lumbricoides*. Simultaneously, biannual deworming campaigns should be implemented for both humans and animals, with public health officials providing deworming tablets to vulnerable groups, such as children, and veterinarians administering broad-spectrum anthelmintics to domestic animals.

To reduce environmental contamination, locally managed composting toilets or sanitary latrines should be introduced to curb open defecation, and designated animal waste collection points to ensure proper disposal. Community workshops and school-based programs are essential for educating residents about parasite transmission, hygiene practices, and safe handling of animal feces. Additionally, contaminated areas should be cleaned through community-driven efforts, and soil treatment protocols such as liming should be applied to reduce parasite egg viability. A local One Health task force, comprising representatives from human, veterinary, and environmental sectors, should oversee the implementation and monitoring of these interventions, using data collected from periodic soil, water, and fecal analyses to refine strategies.

Long-term sustainability can be achieved by promoting enclosed animal husbandry systems and securing funding from NGOs or government agencies to maintain infrastructure improvements and health programs. This integrated approach, emphasizing community engagement and data-driven decisions, can reduce parasitic infections in Sitio Ibayo and improve overall health outcomes [27,28].

Limitations of the study and future directions

The study has several important limitations that must be considered. First, the study was conducted during the dry season. Given that seasonal variations were not accounted for, the diversity and prevalence of parasites are limited and do not fully describe the overall parasite ecology in the area. Second, the focus on Sitio Ibayo, San Mateo, Rizal, limits the generalizability of findings to other communities with differing environmental or socio-economic conditions. The diagnostic methods used, mainly microscopy and flotation techniques, may not be as sensitive as advanced molecular diagnostics, thus missing cryptic or emerging species. To highlight the detection of *Clonorchis* spp. requires molecular identification. While there are few reports of *Clonorchis* spp., this is not common in the country compared to East Asian countries where it is prevalent [29]. On the other hand, the limitations of copro microscopy may have also resulted in underreported prevalence rates of infection with very low intensity undiagnosed.

The study did not directly assess the transmission pathways of zoonotic parasites between animals and humans, thus leaving gaps in understanding zoonotic risks. Longitudinal data are not available, therefore limiting insights into temporal trends in parasite prevalence and intensity. Addressing these limitations will greatly enhance the understanding of parasite ecology and zoonotic disease transmission in future studies.

As a sentinel site for ongoing zoonotic disease surveillance, there should be continuous monitoring of the area's diversity, prevalence, and transmission dynamics of parasites. Advanced diagnostic tools, including molecular methods, should be used to identify emerging or cryptic parasitic species and monitor their evolution over time. Future studies should also assess the effectiveness of integrated interventions in reducing parasitic burden, such as improved sanitation, targeted deworming programs, and community health education. This will expand the scope to include seasonal variations and longitudinal data, thus providing deeper insights into parasite ecology and informing scalable strategies for other similar high-risk communities, aligned with the One Health framework to mitigate zoonotic risks and enhance public health outcomes.

## Conclusions

This study provides critical insights into the diversity and prevalence of parasites in a suburban community in Sitio Ibayo, San Mateo, Rizal, with significant public health and ecological implications. The findings reveal widespread parasitic infections among various animal groups, highlighting notable polyparasitism and zoonotic risks. Dogs and other small animals were identified as key reservoirs for multiple parasites, underscoring their role in sustaining and amplifying parasitic cycles. The detection of zoonotic parasites such as *A. lumbricoides*, *Giardia* spp., and *Entamoeba* spp. indicates the interconnectedness of human, animal, and environmental health, emphasizing the need for an integrated One Health approach. This study further advances scientific knowledge by detailing parasites' prevalence, intensity, and co-infection patterns in different animal hosts within the suburban community. It highlights their role in zoonotic transmission and ecosystem health, providing a baseline for parasitic epidemiology and supporting integrated One Health strategies for disease management in these settings.
